# 0671. Extended extracorporeal lung support in a porcine acute lung injury model. Feasibility and preliminary data

**DOI:** 10.1186/2197-425X-2-S1-P44

**Published:** 2014-09-26

**Authors:** A Bruhn, P Cruces, P Tapia, P Garcia, L Alegria, J Araos, D Soto, D Hurtado, F Rodriguez, M Amthauer, T Salomon, D Rodriguez, ME Rucán, G Castro, B Erranz, R Cornejo, G Bugedo

**Affiliations:** Pontificia Universidad Catolica de Chile, Departamento de Medicina Intensiva, Santiago, Chile; Universidad Andrés Bello, Centro de Investigación de Medicina Veterinaria, Santiago, Chile; Pontificia Universidad Catolica de Chile, Escuela de Kinesiología, Santiago, Chile; Pontificia Universidad Catolica de Chile, Departamento de Ingeniería Estructural y Geotécnica, Santiago, Chile; Clinica Alemana de Santiago, Unidad de Cuidados Intensivos Pediátricos, Santiago, Chile; Universidad de Chile, Departamento de Medicina, Santiago, Chile

## Introduction

During the last years there has been a renewed interest in using extracorporeal membrane oxygenation (ECMO) to treat severe ARDS. ECMO may correct hypoxemia but may also aid in protecting the lungs [1]. However, there is scarce data about the optimal way to ventilate the lungs during ECMO. Experimental models of acute lung injury with ECMO are usually too short.

## Objectives

To develop an extended severe ARDS model supported with ECMO suitable to assess the impact of different ventilatory strategies during ECMO.

## Methods

Eighteen domestic pigs (27-35 kg) were anesthetized, mechanically ventilated (Vt 10 ml/kg, PEEP 5, O2 1.0) and invasively monitored. Thereafter they were randomized into 3 groups: sham, lung injury, and lung injury + ECMO. SHAM animals were ventilated for 24 hours with standard ventilation (Vt 10 ml/kg, PEEP 5, FiO2 1.0 and RR adjusted to keep PaCO2 30 to 50 mmHg) and then euthanized. In the other groups lung injury was induced by saline lavages (30 ml/kg per lavage) performed repeatedly in both supine and prone position until PaO2/FiO2 dropped below 250. They were then subjected to a 2-hour injurious ventilation with PCV, PEEP = 0,Pinsp = 40 cmH2O, RR = 10/min, I:E = 1:1, one hour in prone and the other in supine. After completing lung injury (time 0) animals were subjected to 24 hours of standard ventilation. In the ECMO group, animals were immediately connected to a saline primed-MEDOS Hilite ECMO circuit by inserting a AVALON 23F double-lumen cannula through the external jugular vein. Blood flow was set at 60-70% of cardiac output. Respiratory and hemodynamic data, as well as plasma and BAL samples were collected at times 0,3, 6, 12, 18 and 24h. After euthanizing animals at time 24h tissue samples were extracted from the lungs.

## Results

The two-hit lung injury model resulted in severe hypoxemia, decreased compliance, and with extensive lung inflammation on histology, with only 6/12 animals surviving up to 24h. Lung tissue revealed extensive inflammation. ECMO resulted in improved survival, increased oxygenation, lower pulmonary artery pressures, but no change was observed on compliance. Wet/dry ratio in lung tissue was 4.1±0.9, 7.2±0.1, and 7.2±0.5 in the SHAM, lung injury and lung injury+ECMO groups, respectively. ECMO setup had minimal recirculation with high O2 and CO2 transfer rates. Although protective ventilation was not applied in this feasibility study in pilot experiments we confirmed that the ECMO setup has the potential to provide extended full lung support.

**Figure 1:**
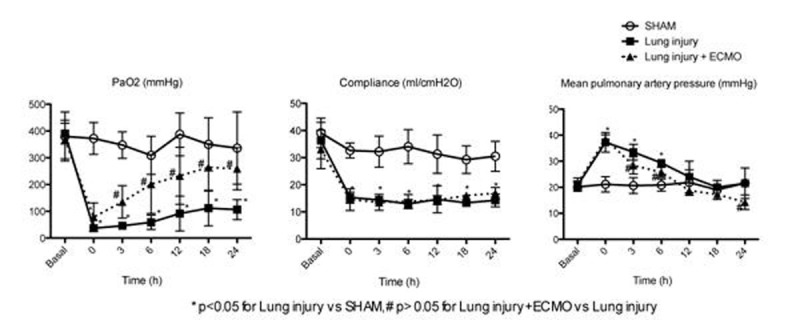
Figure 1

## Conclusions

An extended lung injury model supported with ECMO is feasible. The two-hit model produced a steady compromise in compliance despite partial recovery of hypoxemia.
